# Acute intermittent porphyria complicated with acute pancreatitis: A case report and literature review

**DOI:** 10.1097/MD.0000000000036036

**Published:** 2023-11-17

**Authors:** Cheng Jiao, Wei Liu, Jin-Hui Chen, Jun Guo, Yi-Ming Qiao

**Affiliations:** a Department of General Surgery, Bethune International Peace Hospital, Shijiazhuang, Hebei 050000, China.

**Keywords:** acute intermittent porphyria, acute pancreatitis, case report, hematin

## Abstract

**Rationale:**

Acute intermittent porphyria (AIP) is a rare genetic disorder that affects porphyrin metabolism in the blood. The disease causes defects in specific enzymes in the body, which in turn leads to the accumulation of porphyrin metabolites. Patients may experience abdominal pain, neurological symptoms, muscle pain, and nausea, but it does not directly cause pancreatitis.

**Patient concerns:**

The patient is a young woman, 23 years old, who was admitted to our hospital with intermittent abdominal pain for 2 days, the pain was not fixed, episodic, with no obvious trigger, and 1 day before admission, the patient started to experience nausea and vomiting, with gastric contents as the vomitus, and similar symptoms had occurred many times in the past. Blood amylase 600 U/L, blood sodium 120.6 mmol/L, blood routine, and coagulation function results were normal; abdominal CT showed pancreatic swelling with unclear surrounding fat interstitial, acute pancreatitis was considered. The patient’s urine was dark red, and the results of the qualitative urine porphyrin test were positive.

**Diagnoses:**

AIP complicated with acute pancreatitis.

**Intervention:**

Relief of symptoms, control of pain, correction of electrolyte disturbances, and high-carbohydrate therapy.

**Outcomes:**

The patient was discharged with complete symptomatic relief after 10 days of high-carbohydrate therapy.

**Lessons:**

AIP complicated with acute pancreatitis is very rare. Treatment of AIPs aims to control acute attacks and prevent potential triggers.

## 1. Introduction

Porphyria are a group of inherited diseases caused by a disorder of porphyrin metabolism in the body.^[1]^ Porphyrins are chemicals produced in the body that are involved in oxygen transport by combining with iron ions in the blood to form hemoglobin.^[2]^ There are 2 main types of porphyria: acute and chronic. Acute porphyria are abnormalities in porphyrin metabolism due to defects in specific enzymes in the body; the most common type is acute intermittent porphyria (AIP).^[3,4]^ Attacks of the disease are associated with abdominal pain, vomiting, and neurological symptoms, and may be misdiagnosed as other acute abdominal conditions. In severe cases, the disease may lead to damage to the heart, nervous system, and other organs, and may even be life-threatening.^[5]^ In this article, we report a rare case of AIP complicated with acute pancreatitis and analyze relevant literature.

## 2. Case presentation

A 23-year-old woman presented to our hospital with intermittent abdominal pain for 2 days. The pain was not specific to any location and occurred in paroxysms without an obvious trigger. One day before admission, the patient began experiencing nausea and vomiting, with vomitus consisting of gastric contents. Similar symptoms had occurred in the past, but the patient denied any history of special diseases. On physical examination at admission, the patient showed mild tenderness throughout the abdomen without rebound tenderness or other positive signs. Laboratory tests revealed elevated serum amylase levels (600 U/L) and hyponatremia (serum sodium level of 120.6 mmol/L). Blood routine examination and coagulation function were normal. Abdominal CT revealed pancreatic edema and unclear surrounding fat interspaces, indicative of acute pancreatitis.

**Figure 1.**
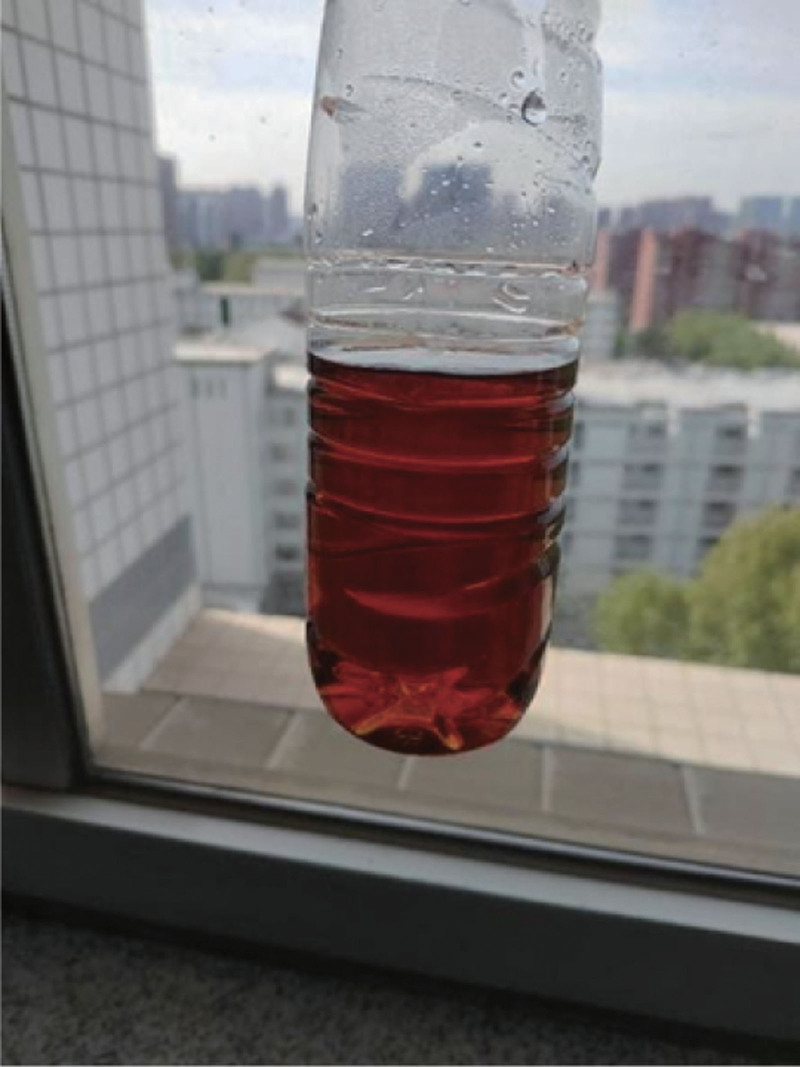
The color of urine.

The patient was diagnosed with acute pancreatitis and hyponatremia. Treatment included inhibition of pancreatic secretion, fasting, gastrointestinal decompression, intravenous nutrition, sodium supplementation (5 mL/h of 10% hypertonic saline), and analgesia. However, the abdominal pain did not significantly improve, although the nausea and vomiting subsided somewhat. reexamination showed a decrease in serum amylase to 200 U/L and an increase in serum sodium to 122.6 mmol/L. It was also observed that the patient’s urine was dark red in color (Fig. 1), which became darker after standing for a while.

There was a high suspicion of AIP combined with acute pancreatitis in the patient. A urine porphyrin qualitative test was positive, and after obtaining consent from the patient and her family, blood samples were taken from the patient and her parents for genetic testing. The results are shown in Table 1, which revealed a mutation in the HMBS gene and confirmed the diagnosis of AIP.

**Table 1:** The detected genes.

Gene	Chromosomal location	cDNA level	Status	Variant classification	Father	Mother
HMBS	chr11:118960419	NM_000190.4:c.293A>C (p.Lys98Thr)	Heterozygous	Suspected	Carried the heterozygous mutation	Not detected

After the definitive diagnosis of AIP, a follow-up blood amylase test was normal and abdominal CT showed significant improvement in pancreatitis. Considering drug availability, the patient was given a high-carbohydrate diet (300 g/d glucose) and received opioid analgesics for pain management. Blood amylase levels were monitored and remained normal during treatment. After 10 days of high-carbohydrate therapy, the patient’s symptoms completely resolved, and she was discharged from the hospital.

## 3. Discussion

AIP is a rare autosomal dominant genetic disease caused by a deficiency of the third enzyme in heme biosynthesis, hydroxymethylbilane synthase (HMBS), leading to the accumulation of the porphyrin precursors delta-aminolevulinic acid (ALA), and porphobilinogen (PBG). The toxic effects of these porphyrin precursors can result in a range of symptoms, with abdominal pain as the predominant feature, accompanied by various neurological and psychiatric manifestations.^[6]^ In addition to AIP, there are 3 other genetic porphyrias that share a similar clinical presentation and are collectively referred to as acute hepatic porphyrias. These include delta-aminolevulinic acid dehydratase deficiency porphyria, hereditary coproporphyria, and variegate porphyria. Among these subtypes, AIP is the most common.^[7]^

AIP is a rare autosomal dominant genetic disease characterized by recurrent acute onset. AIP attacks can be triggered by various factors such as surgery, certain medications, radiation exposure, pregnancy, menstruation, infection, and fasting.^[8]^ In young women, hormonal fluctuations and weight loss are identified as the primary triggers according to several studies.^[5]^

The clinical features of AIP primarily include abdominal pain, neurological dysfunction, and psychiatric abnormalities.^[9]^ Acute abdominal pain is the most common and earliest symptom of AIP, with over 95% of Chinese patients presenting with this symptom.^[7,10]^ AIP-induced abdominal pain is typically severe, persistent, and diffuse, accompanied by constipation, bloating, nausea, vomiting, and occasionally intestinal obstruction. Neurological and psychiatric abnormalities are also hallmark features of AIP acute attacks. These can be classified as central nervous system symptoms, peripheral nervous system symptoms, autonomic nervous system symptoms, and psychiatric symptoms. Central nervous system symptoms can manifest as seizures, altered consciousness, limb paralysis, respiratory failure, and coma. Peripheral nervous system symptoms often involve motor neuropathy and sometimes sensory abnormalities.^[11]^ Autonomic nervous system symptoms can cause hypertension, tachycardia, sweating, electrolyte imbalances, and hyponatremia.^[12]^ The mechanism of tachycardia may be related to sympathetic overactivity and increased catecholamine levels, while hyponatremia may result from hypothalamic impairment, abnormal antidiuretic hormone secretion, vomiting, inadequate sodium intake, and increased renal sodium excretion. Psychiatric symptoms can include emotional lability, depression, and anxiety, and a few patients may experience auditory or sensory hallucinations.

AIP is often accompanied by liver dysfunction, significantly increasing the risk of cirrhosis, liver cell carcinoma, and kidney failure.^[5,13]^ Early diagnosis and standardized treatment are crucial for improving long-term prognosis. The diagnosis of AIP mainly relies on clinical manifestations and enzyme assays, with significant increases in urine ALA and PBG during acute attacks. Genetic testing is the gold standard for AIP diagnosis, but it is not yet widely available in most medical institutions, and the testing process is time-consuming, making timely diagnosis challenging. During acute attacks, patients’ urine is often red or dark red due to the presence of large amounts of porphobilinogen, which turns into a characteristic burgundy color after exposure to sunlight, providing an important diagnostic clue.

In terms of treatment, timely removal of trigger factors and symptomatic treatment are vital for controlling AIP attacks and improving prognosis. According to some studies, the use of ovarian function suppression drugs to block menstruation can effectively prevent AIP attacks in young women.^[14,15]^ High-carbohydrate therapy is the primary treatment for acute AIP attacks. Increased carbohydrate intake can interfere with ALAS1 transcription and inhibit the accumulation of related products in the body, thereby improving symptoms.^[16]^ Glucose can also stimulate insulin secretion, directly inhibiting ALAS1 expression.^[17,18]^ In the acute phase of AIP attacks, a high-carbohydrate diet (300–500 g/d) is recommended. Heme is currently the only effective drug for AIP treatment. Heme not only downregulates ALAS1 transcription through negative feedback but also reduces hepatic ALAS1 levels by interfering with mRNA stability or blocking mature enzymes from entering the mitochondria.^[19]^ However, this drug is expensive, and its availability is limited in China due to the difficulty in diagnosing AIP. Therefore, it is not suitable as an experimental treatment for highly suspected patients. For acute pain caused by AIP, nonsteroidal anti-inflammatory drugs and opioids are effective, but their oral efficacy is poor. Gene therapy is currently the main research direction and hot topic in AIP treatment, mainly including gene supplementation therapy and gene silencing therapy.^[20]^ The former involves introducing wild-type HMBSmRNA into liver cells to increase PBGD protein levels significantly, while the latter involves introducing specific small interfering RNA (siRNA) into liver cells to silence ALAS1 mRNA, reducing ALA and PBG production.^[13,21]^ Givosiran is a representative drug that has been studied in clinical trials and can rapidly and continuously reduce ALAS1 mRNA levels, decrease heme precursor levels, and reduce the incidence of acute attacks, with good clinical prospects.^[22]^

## 4. Conclusion

In this case, the patient presented with symptoms of acute pancreatitis, and relevant characteristics of the case met the diagnostic criteria for acute pancreatitis. Similar cases with AIP as the underlying cause are rare in China. AIP has complex and diverse clinical manifestations and lacks specificity, leading to high misdiagnosis and missed diagnosis rates of up to 70%.^[23]^ Therefore, caution should be exercised when using high-carbohydrate therapy to avoid secondary damage to pancreatic function. Some studies have reported that some patients were diagnosed with AIP after negative exploratory surgery, indicating that clinicians should be aware of the possibility of AIP acute attacks when dealing with patients whose primary symptoms are acute abdominal pain, while also considering surgical indications.

## Author contributions

**Conceptualization:** Cheng Jiao, Wei Liu, Jin-Hui Chen, Jun Guo, Yi-Ming Qiao.

**Data curation:** Wei Liu, Jun Guo, Yi-Ming Qiao.

**Funding acquisition:** Cheng Jiao, Wei Liu, Jun Guo, Yi-Ming Qiao.

**Methodology:** Cheng Jiao, Wei Liu, Jun Guo, Yi-Ming Qiao.

**Project administration:** Cheng Jiao.

**Resources:** Cheng Jiao, Wei Liu, Jin-Hui Chen, Jun Guo, Yi-Ming Qiao.

**Software:** Cheng Jiao.

**Supervision:** Cheng Jiao, Wei Liu, Jun Guo.

**Validation:** Cheng Jiao, Jin-Hui Chen, Yi-Ming Qiao.

**Writing – original draft:** Cheng Jiao.

**Writing –review & editing:** Wei Liu.
